# Treatment Adherence and Its Determinants in the Geriatric Population With Non-communicable Diseases Living in Urban Slums of Pune: An Analytical Cross-Sectional Study

**DOI:** 10.7759/cureus.94645

**Published:** 2025-10-15

**Authors:** Aditya Sharma, Sanjivani V Patil, Rupeshkumar B Deshmukh, Saibal Adhya, Varsha Vaidya

**Affiliations:** 1 Community Medicine, Bharati Vidyapeeth (Deemed to be University) Medical College, Pune, IND

**Keywords:** elderly, geriatric population, medication, non-communicable diseases, pune, treatment adherence, urban slums

## Abstract

Background/Purpose: Treatment adherence is crucial for managing noncommunicable diseases (NCDs), improving quality of life, and preventing complications, especially among the geriatric population. It remains a significant challenge in urban slums where out-of-pocket expenses contribute to non-adherence; hence, this study aims to assess treatment adherence.

Methods: Under the field practice area of a UHTC (urban health training center), a cross-sectional analytical study was conducted in the slums. A two-stage sampling method was used to select study participants, consisting of geriatric population individuals aged 60 years. Data was collected with the help of a questionnaire. To assess medication adherence, the General Medication Adherence Scale (GMAS) was used. Statistical analyses were performed using IBM SPSS Statistics for Windows, Version 29 (Released 2024; IBM Corp., Armonk, New York, United States). The associations were calculated using odds ratios (ORs) and 95% confidence intervals (CIs).

Results: A total of 122 participants were included, comprising 69 (56.6%) female patients and 53 (43.4%) male patients, with 78 (63.9%) residing in joint families. The most common NCDs were type 2 diabetes mellitus (45.9%) and hypertension (39.3%). Overall, 80.3% demonstrated good adherence, while 19.7% showed poor adherence. Poor adherence was significantly associated with the occupation of the household head (OR = 3.97; 95% CI: 1.56-10.08; p = 0.003), and non-working participants also had higher odds (OR = 4.40; 95% CI: 0.97-19.96; p = 0.054). Lifestyle factors such as diet, exercise, and tobacco or alcohol use were not significantly associated. Major barriers included low awareness (42.6%) and poor socioeconomic status (38.5%).

Conclusion: This study among the geriatric population observed high medication adherence. It is essential to raise awareness about NCD management. Addressing barriers can significantly increase treatment adherence.

## Introduction

Non-communicable diseases (NCDs), sometimes referred to as chronic diseases, are frequently caused by a confluence of environmental, behavioral, genetic, and physiological factors. The most prevalent forms of NCDs include cardiovascular conditions (like heart attacks and stroke), cancer, chronic respiratory conditions (like asthma and chronic obstructive pulmonary disease), and type 2 diabetes [1]. The risk factors for NCDs encompass modifiable behaviors such as tobacco use, physical inactivity, excessive alcohol consumption, and unhealthy dietary habits, as well as non-modifiable factors like advancing age. Low- and middle-income countries account for three-quarters of these deaths every year, and globally, NCDs are responsible for nearly 71% of total mortality [1]. The presence of two or more chronic diseases is called multimorbidity. It is increasing globally and India faces numerous health challenges due to NCDs and an aging population [2]. A healthy lifestyle and regular follow-ups are key factors in NCD treatment, alongside medication adherence [3]. In order to address the long-term care requirements of the elderly population with physical or mental impairments, concerted efforts are needed to establish integrated health, social, and community-based services that promote their quality of life, safeguard their independence, and reduce the burden on families and caregivers [3]. The global population is rapidly aging as a result of longer life expectancy and lower fertility. World Population Prospects 2019 projects that by 2050, 1 out of every 6 people worldwide will be over the age of 65 [3]. In the 2001 census, 77 million was the geriatric population in India; by 2011, it had increased to 96 million, and by 2050, it is projected to reach 301 million [4]. Globally, the proportion of elderly individuals is increasing as a consequence of rising life expectancy, which in turn has contributed to a higher burden of age-related health concerns within this population group. This demographic transition carries wide-ranging implications, including an elevated prevalence of chronic NCDs, increased demand for long-term care and rehabilitation services, escalation of healthcare expenditures, and heightened strain on existing health systems and social security frameworks. Furthermore, it raises challenges related to functional dependency, caregiver burden, and the necessity for comprehensive policies that foster healthy aging, social integration, and the financial sustainability of health and welfare programs [4]. NCDs are no longer limited to urban and affluent populations; epidemiological and demographic shifts have made them a significant risk in low-income settings. Adherence to treatment is critical for increasing quality of life and preventing NCD complications. The World Health Organization estimates that nearly 50% of patients with chronic illnesses in developed countries do not adhere to prescribed treatment regimens, with adherence rates worldwide ranging from 40% to 75% depending on the setting [5]. This variability reflects challenges such as polypharmacy, comorbidities, limited health literacy, and socioeconomic barriers. In the Indian context, Angadi et al. (2020) reported suboptimal adherence among elderly individuals with chronic conditions in urban South India, consistent with other national studies that show only 40-60% of older adults achieve adequate adherence to long-term therapies [6]. Non-compliance has been linked to a variety of factors, including medication-related issues such as side effects, cost, inadequate guidance, and polypharmacy. Age, cognitive and sensory impairments, and other co-existing health conditions are all factors that can influence the patient’s overall health management, functional independence, and quality of life [6]. According to research, failure to take prescribed medications leads to disease progression, worsening symptoms, increased medical interventions, more frequent hospitalizations, and an increased risk of mortality [2-4,6].

The geriatric population faces heightened vulnerability, and inadequate compliance with treatment regimens constitutes a critical obstacle to achieving better health outcomes and overall quality of life. Moreover, limited region-specific studies exist, particularly for urban slums, where financial hardship and out-of-pocket expenses are major contributors to medication non-adherence. Hence, the present study is planned to assess treatment adherence, defined as the extent to which elderly individuals follow their prescribed medication and lifestyle regimens as recommended by healthcare providers, and its determinants for NCDs among the geriatric population residing in urban slums of Pune.

## Materials and methods

Study design

This was an analytical cross-sectional study conducted from August 2024 to January 2025 among geriatric patients aged 60 years and above residing in urban slums within the field practice area of a medical college (Bharati Vidyapeeth (Deemed to be University) Medical College) in Pune, Maharashtra.

Ethical considerations

The study protocol was approved by the Institutional Ethical Committee of the central research institute of the medical college under approval number BVDUMC/IEC/76 dated September 28, 2024. Informed written consent was obtained from all study participants.

Study setting and participants

The study population included all geriatric residents (≥ 60 years) who had been living in the study area for more than six months and had been diagnosed or confirmed with NCDs by a general practitioner in the previous six months. Exclusion criteria included geriatric individuals who were critically ill or whose households were locked during two consecutive visits.

Sample size and sampling method

The sample size was calculated using the formula N = 4pq/d² [7], where p was 58.5% (medication adherence from a reference study). With an allowable error of 8.77% of p (15% of the proportion) and a 95% confidence level, the minimum required sample size was 122.

A two-stage sampling method was employed. In the first stage, one ward was randomly selected from the three wards in the field practice area. In the second stage, one administrative block within the selected ward was chosen randomly. A sampling frame comprising the list of all elderly residents in the study area (N = 1000) was obtained. From this frame, 470 households were screened using simple random sampling to achieve the required sample size. If a selected household did not have an eligible participant or was unavailable, the next household on the list was included. In households with more than one eligible elderly participant, only one individual was selected randomly.

Questionnaire and data collection

Data were collected using a pre-designed, semi-structured questionnaire. The initial draft of the tool was prepared in English after an extensive review of existing literature on medication adherence and barriers to adherence in geriatric populations. The draft questionnaire was then reviewed for content validity by two subject experts-one faculty member from the Department of Medicine and another from the Department of Community Medicine at different medical colleges. Their feedback was incorporated to improve clarity, relevance, and comprehensiveness of the items. For linguistic accuracy, the finalized version was translated into Marathi, followed by back-translation into English, ensuring that the semantic meaning of each item remained intact. It covered the following.

Socio-demographic Variables

Information on socio-demographic characteristics was collected using a pretested structured questionnaire. Data included age, sex, type of family, marital status, education, occupation, and monthly family income. Socioeconomic status was assessed using the modified Kuppuswamy scale (2024) [8]. Additional details such as education and occupation of the head of the family, current source of income (e.g., pension, job, rent, relatives, NGOs), and level of financial dependence (independent or dependent) were also recorded.

Morbidity Profile

Participants were asked to report their diagnosed NCDs and duration of illness. Information on comorbidities, preferred source of healthcare (government, private, or both), and living arrangements (with family or alone) was collected. Family history of common NCDs such as type 2 diabetes mellitus, hypertension, stroke and chronic lung disease was also obtained.

Medication Details

Data regarding medication use included current medications prescribed for NCDs, number of medications (monotherapy, dual therapy, or polytherapy), dosage, and frequency. Participants were asked where they obtained their medications (public or private facilities) and whether they adhered to prescribed regimens. Additional information on any side effects experienced, changes in medication use (altering dose or frequency), and frequency of follow-up visits as advised by the treating physician was also collected.

Barriers to Medication Adherence

Barriers to adherence were assessed using a structured adherence scale that explored factors across multiple domains: (i) Patient-related factors: forgetfulness, being busy, or stopping medication when feeling well; (ii) Therapy-related factors: side effects, regimen complexity, or addition of new medicines; (iii) Healthcare system-related factors: irregular follow-up, inadequate counselling, or limited access to healthcare facilities; (iv) Social and economic factors: cost of medicines, out-of-pocket expenditure, and lack of family or community support.

Medication adherence refers to adherence to prescribed medications among the geriatric population, while treatment adherence encompasses lifestyle and behavioral components such as alcohol and tobacco use, physical activity, and adherence to dietary advice that may influence overall adherence patterns.

Medication adherence was assessed using the 11-item validated General Medication Adherence Scale (GMAS), which has a maximum score of 33. The scale evaluates three domains: (i) patient behavior-related non-adherence, (ii) comorbidity and pill burden-related non-adherence, and (iii) cost-related non-adherence. Each item was rated on a four-point Likert scale. Based on the total score, adherence was categorized as good adherence (>27) and poor adherence (≤27). Specifically, the scale exhibited a sensitivity greater than 75% and a specificity exceeding 80% in assessing medication adherence among patients with chronic illnesses. These values indicate that the GMAS is effective in accurately identifying both adherent and non-adherent patients, making it a reliable tool for adherence assessment in clinical and research settings [9].

Data collection was done through interviews, using prescriptions and investigation reports for verification where available. In case of cognitively impaired participants, caregivers were interviewed.

Statistical analysis

Data were coded and entered into Microsoft Excel, and analysis was performed using IBM SPSS Statistics for Windows, Version 29 (Released 2024; IBM Corp., Armonk, New York, United States). Descriptive statistics were presented as percentages. Chi-square test/Fisher’s exact test was used to compare categorical variables by the adherence level. Odds ratios (ORs) with 95% confidence intervals (CIs) were calculated to examine associations between medication adherence and socio-demographic/other factors. A p-value of <0.05 was considered statistically significant.

## Results

The present study examined the proportion of treatment adherence for NCDs among geriatric patients living in Pune's urban slums, as well as the determinants and barriers to medication compliance. The study included 122 geriatric patients with one or more chronic diseases. Table 1 shows the distribution of study participants based on their sociodemographic profile.

**Table 1:** Distribution of study participants according to the sociodemographic profile (n=122) SES 2024: Socio-Economic Scale - Modified Kuppuswamy 2024 scale *The head of the family was defined as the adult member recognized by the household as the primary decision-maker, whose education, occupation, and income were used to determine the socioeconomic status according to the Kuppuswamy 2024 scale.

Variable	Category	Frequency and percentage n (%)
Age group (in years)	60-69	71 (58.19)
	70-79	41 (33.60)
	80-89	10 (8.19)
Sex	Male	53 (43.47)
	Female	69 (56.68)
Type of family	Joint	78 (63.98)
	Nuclear	26 (21.34)
	Others	18 (14.82)
Marital status	Married	61 (50.00)
	Widow	46 (37.76)
	Widower	15 (12.35)
Color of the ration card	Orange	100 (82.00)
	White	4 (3.38)
	Yellow	18 (14.84)
Education of the head of the family*	≤ Primary	60 (49.18)
	> Primary	62 (50.81)
Occupation of the head of the family	Elementary occupation	68 (55.76)
	Skilled worker	10 (8.24)
	Unemployed	44 (36.15)
SES 2024	Lower middle (III)	10 (8.22)
	Upper lower (IV)	80 (65.67)
	Lower(V)	32 (26.23)
Education of the study participant	≤ Primary	90 (73.77)
	> Primary	32 (26.22)
Occupation of the study participant	Not working	92 (75.46)
	Working	30 (24.64)
Financial dependence	Dependant	82 (67.21)
	Independent	40 (32.78)

The average age of study participants ranged from 60 to 88 years and was 67.88±7.24 (mean ± SD) years. Among them, 56.55% were females. The majority of participants lived in joint families (78; 63.93%) and the majority of them possessed orange ration card (82.00%; n=100). Most of the participants (80; 65.67%) belong to the upper lower class (IV) per Modified Kuppuswamy 2024 scale. Monthly family income ranged from 1500 to 40000.

Table 2 shows the morbidity profile of study participants which shows that most of the participants were on monotherapy (77; 63.11%). Hypertension was the most common NCD (89; 72.95%), followed by type 2 diabetes mellitus (58; 47.54%). Among the participants, 98 participants (80.33%) had good medication adherence. Table 3 shows the distribution of study participants according to reasons for medication non-adherence; 24 (19.67%) had poor medication adherence and the most common reasons among them were found to be non-affordability of medications (22.73%) and being unaware of medication adherence (26.15%).

**Table 2:** Morbidity profile of study participants (n=173*) *Indicates multiple responses Monotherapy refers to the use of a single medication to manage a specific chronic condition or non-communicable disease (NCD). Dual therapy involves the use of two medications concurrently to control the disease, either from the same therapeutic class or different classes. Polytherapy (or polypharmacy) denotes the use of three or more medications simultaneously for the management of one or multiple chronic conditions.

Variable	Category	Frequency and percentage n(%) *
Type of morbidity	Type 2 diabetes mellitus	58 (47.54)
	Hypertension	89 (72.95)
	Bronchial asthma	14 (11.47)
	COPD	4 (3.27)
	CVA	8 (6.55)
Type of Therapy	Monotherapy	77 (63.11)
	Dual therapy	36 (29.50)
	Polytherapy	9 (7.37)

**Table 3:** Reasons for non-adherence

Reason for Non-adherence	Percentage (%)
Not aware	26.15
Not affordable	22.73
Out of pocket expenses	20.24
Others	13.43
Non-availability	10.12
Non-accessibility	7.67

Medication adherence was poor in 24 (19.67%) participants, while 98 (80.30%) reported good adherence (Table 4). Type of therapy (monotherapy vs. others) was not significantly associated with adherence (OR = 0.63; 95% CI: 0.25-1.55; p = 0.31). Socio-demographic variables such as age ≤70 years (19 (79.17%) vs. 70 (71.43%); OR = 1.52; 95% CI: 0.52-4.47), sex (female: 13 (54.17%) vs. 56 (57.14%); OR = 0.89; 95% CI: 0.36-2.17), type of family (joint: 12 (50.00%) vs. 66 (67.35%); OR = 0.48; 95% CI: 0.19-1.20), marital status, education of head of family, socio-economic status, education of the participant, and financial dependence did not show significant associations with medication adherence. However, occupation of the head of the family was a significant determinant. Participants whose household head was unemployed had nearly four times higher odds of poor adherence compared to those with employed/skilled household heads (15 (62.50%) vs. 29 (29.59%); OR = 3.97; 95% CI: 1.56-10.08; p = 0.003). Similarly, occupation of the study participant showed a trend toward significance: non-working participants had higher odds of poor adherence (22 (91.67%) vs. 70 (71.43%); OR = 4.40; 95% CI: 0.97-19.96; p = 0.054). Treatment adherence also included lifestyle modifications (Table 4). Among participants, 117 (95.90%) reported abstaining from alcohol, 78 (63.93%) abstained from tobacco, 37 (30.33%) followed dietary advice, and 24 (19.67%) reported regular exercise. However, these behaviors did not show statistically significant associations with medication adherence. Poor adherence was slightly higher among those who did not follow dietary advice (19 (79.17%) vs. 66 (67.35%); OR = 0.54; 95% CI: 0.18-1.59), did not exercise (20 (83.33%) vs. 78 (79.59%); OR = 0.78; 95% CI: 0.24-2.54), consumed tobacco (11 (45.83%) vs. 33 (33.67%); OR = 0.60; 95% CI: 0.24-1.48), and consumed alcohol (1 (4.17%) vs. 4 (4.08%); OR = 0.98; 95% CI: 0.10-9.18), but none were statistically significant. Overall, treatment adherence among the geriatric population in urban slums was more strongly influenced by socio-demographic determinants, particularly occupation of the head of family, than by lifestyle or therapy-related factors.

**Table 4:** Association of medication adherence with socio-demographic variables (n=122) Alcohol consumption is defined as the intake of any beverage containing ethanol, including beer, wine, spirits, or locally brewed alcoholic drinks, by an individual over a specified period. For measurement purposes, it can be categorized based on frequency and quantity: (i) Never drinker: Individuals who have never consumed any alcoholic beverage in their lifetime; (ii) Former drinker: Individuals who have consumed alcohol in the past but have abstained for at least the last 12 months; (iii) Current drinker: Individuals who have consumed any form of alcoholic beverage within the past 12 months; (iv) Binge drinking: Consumption of ≥5 standard drinks for men or ≥4 standard drinks for women on a single occasion, as per WHO guidelines. Consumption may further be quantified in terms of frequency (daily, weekly, monthly, occasional) and volume (number of standard drinks per occasion), to assess patterns of use and potential health risks. Categorization of exercise: Based on Frequency: (i) Regular exercisers: Individuals performing physical activity ≥3 times per week; (ii) Occasional exercisers: Individuals performing physical activity 1–2 times per week; (iii) Non-exercisers: Individuals performing physical activity less than once per week or not at all. Categorization of dietary advice: (i) Adherent: Individuals who report following the recommended diet most of the time or always (≥80% of the time), including consumption of prescribed meals, portion control, limiting salt, sugar, or fat, and avoiding prohibited foods; (ii) Partially adherent: Individuals who follow the dietary advice sometimes (50–79% of the time); (iii) Non-adherent: Individuals who rarely or never follow the recommended diet (<50% of the time).

Variable	Category	Medication adherence		Total (%)	p-Value	OR(95%CI)	p-value	
		Poor adherence n=24 (19.67%)	Good adherence n=98 (80.30%)					
Age (in years)	≤70	19(79.17)	70(71.43)	89(72.95)	0.44	1.52(0.52-4.47)	0.45	
	>70	5(20.83)	28(28.57)	33(27.05)		1		
Sex	Female	13 (54.17)	56 (57.14)	69 (56.56)	0.9	0.89(0.36-2.17)	0.79	
	Male	11 (45.83)	42 (42.86)	53 (43.44)		1		
Type of family	Joint	12 (50.00)	66 (67.35)	78 (63.93)	0.11	0.48(0.19-1.20)	0.12	
	Nuclear/Others	12(50.00))	32(32.65)	44(36.07)		1		
Marital status	Married	13(54.17)	48(48.98)	61(50.00)	0.65	1.23(0.50-3.01)	0.65	
	widow/Other	11(45.83)	50(51.02)	61(50.00)		1		
Education of the HOF	≤Primary School	13 (54.17)	47 (47.96)	60 (49.18)	0.59	1.28(0.52-3.14)	0.59	
	>Primary School	11 (45.83)	51 (52.04)	62 (50.82)		1		
Occupation of the HOF	Unemployed	15 (62.50)	29 (29.59)	44 (36.07)	0.003	3.97(1.56-10.08)	0.003	
	Elementary occupation/Skilled	9(37.5)	69(70.40)	78(63.93)		1		
SES 2024	Upper Lower (IV)	16 (66.67)	64 (65.31)	80 (65.67)	0.90	1.06(0.41-2.73)	0.90	
	lower middle (III)+Lower(V)	8(33.33)	34(34.69)	42(34.43)		1		
Education of the study participant	Primary+ Illiterate	15(62.50)	75(76.53)	90(73.77)	0.16	0.51(0.19-1.32)	0.17	
	Middle +High School	9(37.50)	23(23.47)	32(56.23)		1		
Occupation of the study participant*	Not working	22(91.67)	70(71.43)	92(75.41)	0.06	4.40(0.97-19.96)	0.054	
	Working	2(8.33)	28(28.57)	30(24.59)		1		
Financial dependence	Dependent	17 (70.83)	65 (66.33)	82 (67.21)	0.67	1.23(0.46-3.27)	0.67	
	Independent	7 (29.17)	33 (33.67)	40 (32.79)		1		
Medication	Monotherapy	13(54.17)	64(65.31)	77(63.11)	0.31	0.63(0.25-1.55)	0.31	
	Other	11(45.83)	34(34.69)	45(36.89)		1		
Do you consume alcohol*	No	23(95.83)	94(95.92)	117(95.90)	0.99	0.98(0.10-9.18)	0.98	
	Yes	1(4.07)	4(4.08)	5(4.10)		1		
Do you consume tobacco	No	13(54.17)	65(66.33)	78(63.93)	0.27	0.60(0.24-1.48)	0.27	
	Yes	11(45.83)	33(33.67)	44(36.07)		1		
Do you exercise	Yes	4 (16.67)	20 (20.41)	24 (19.67)	0.68	0.78(0.24-2.54)	0.68	
	No	20 (83.33)	78 (79.59)	98 (80.33)		1		
Do you follow diet advice	Follow	5 (20.83)	32 (32.65)	37 (30.33)	0.26	0.54(0.18-1.59)	0.26	
	Don’t follow	19 (79.17)	66 (67.35)	85 (69.67)		1		
* Indicates Fisher's Exact Test								

## Discussion

In the present study, poor medication adherence was observed in 19.7% of participants, a finding close to that of Punnapurath et al. (18%) [10]. In contrast, several studies have documented considerably higher levels of poor adherence: Angadi et al. reported 57.5% among the geriatric population [6], a rural Puducherry study noted 32.7% among NCD patients [11], a Tamil Nadu study found 57.6% [12], and Polat et al. reported 45.3% in their geriatric cohort [13]. These differences may be attributed to variations in study settings, socio-demographic profiles, health system accessibility, and the availability of counseling and support services, underscoring the importance of contextual factors in influencing treatment adherence.

Similarly, Jiaming et al. reported low adherence in 36.2% of their elderly Chinese cohort with multiple chronic conditions [14]. The higher medication adherence observed in our study may be attributed to the urban setting, where healthcare accessibility is better and where regular NCD screening of the geriatric population is carried out by a local non-government organization (NGO). Our study found that female participants had higher adherence than male participants, which could be due to better health awareness, greater involvement in family and household health, more frequent contact with the health system, and the stronger influence of community health workers. However, this association was not statistically significant (p = 0.90), a finding also supported by Punnapurath et al. (p = 0.55) [10].

Reasons for non-adherence in our study included irregular medication use, with 32.78% (n = 40) reporting poor adherence; the most common reasons were non-affordability of medications (22.73%) and lack of awareness about medication adherence (26.15%), followed by lower socio-economic status. Angadi et al. [6] similarly identified regimen complexity and cost as key deterrents, while also emphasizing the importance of follow-up practices and patient education in ensuring long-term adherence.

Das et al. [15] further highlighted the challenges of polypharmacy in Indian urban communities, reporting a prevalence of 33.7% and self-medication in 19.7% of older adults, with risk factors such as multiple comorbidities (aOR 2.5), recent hospitalization (aOR 4.6), transition of care (aOR 3.3), and living alone (aOR 4.5). Notably, 65.3% lacked knowledge regarding self-medication, 50% were unaware of risks, and 40.7% engaged in unsafe practices, with NSAIDs (59%) and paracetamol (42%) being the most commonly used drugs. These findings reinforce that multimorbidity and polypharmacy contribute not only to complexity in medication regimens but also to unsafe practices and heightened risk of poor adherence. Alhabib et al. [16] reported similar morbidity patterns among elderly patients in Riyadh, where hypertension and diabetes were observed in 61.7% and 38.2% of participants, respectively, which is consistent with our study. This high burden of multimorbidity is associated with lower quality of life and increased complexity in healthcare needs.

In our study, the most common barriers to adherence were non-affordability, a lack of family support, and the presence of multiple comorbidities. These findings highlight the multifaceted nature of non-adherence, particularly in populations with limited financial or social resources. Punnapurath et al. reported that self-discontinuation of medication was a major concern, which could be attributed to inadequate patient education or counselling [10]. Similarly, Angadi et al. emphasized the importance of physician-patient interaction and communication, as well as regimen complexity and side effects [6]. Together, these findings highlight the importance of patient-centered interventions that address both social and behavioral determinants of adherence. As this was a cross-sectional study based on self-reported data, it is subject to recall and social desirability bias, with limited ability to establish causality.

Limitations

First, as a cross-sectional design, it captures associations at a single point in time and cannot establish causal relationships between predictors and medication adherence. Second, self-reported adherence measures may be subject to recall bias and social desirability bias, potentially leading to overestimation of adherence rates. Although the use of a validated tool (GMAS) strengthens reliability, responses may still be influenced by participants’ perception and memory. Third, exclusion of critically ill individuals and locked households may have introduced a selection bias, possibly limiting generalizability to the most vulnerable geriatric groups. Fourth, the study relied on field practice area data within an urban slum of a single medical college, which may not be representative of rural or diverse urban populations. Fifth, while the questionnaire was validated by experts and pre-tested, comprehensive psychometric validation, such as test-retest reliability or construct validity assessment, was not undertaken. Lastly, although the study assessed multiple barriers to adherence, potential unmeasured confounders such as psychosocial factors, cultural beliefs, and physician-related determinants could not be fully explored. These limitations should be considered when interpreting the findings, and future longitudinal, multicentric studies with mixed-method approaches are recommended to provide deeper insights.

## Conclusions

The proportion of medication adherence was found to high among geriatric urban slum population with females showing better adherence than men. However, socio-economic challenges, financial dependency, and affordability remain critical barriers to treatment adherence. A significant relationship was found between the occupation of the family head and medication adherence, indicating the importance of household-level economic stability in treatment continuity. While medication adherence improved, adherence to lifestyle changes was low, highlighting the need for comprehensive, patient-centered care. Critical strategies for improving adherence in diverse populations include making medications more affordable and accessible, and promoting effective communication between healthcare providers and patients. The study focuses on need for population-specific measure to improve medication adherence.

## Appendices

Proforma in English

Serial no -

Name of the participant

Date of interview

 Address

Section A: Sociodemographic Details

Age (In completed years)

Sex - Male /Female /Others

Type of family - Joint/ Nuclear/ Others (Specify)

Marital status - Single /married/separated/divorced/widowed/widower

Color of Ration card - White / yellow / saffron 

Education of the head of the family - 

Profession or honors - (score 7)

Graduate - (score 6)

Intermediate or diploma -(score 5)

High school certificate - (score 4)

Middle school certificate - (score 3)

Primary school certificate -(score 2)

Illiterate - (score 1)

Occupation of head of family

Legislators, senior officers, and managers - (score 10)

Professionals - (score 9)

Technicians and associate professionals - (score 8)

Clerks - (score 7)

Skilled workers and shop and market sales workers - (score 6)

Skilled agricultural and fishery workers - (score 5)

Craft and related trade workers - (score 4)

Plant and machine operators and assemblers - (score 3)

Elementary occupation - (score 2)

Unemployed - (score 1)

Monthly family income (INR)-   ……………….. (INR)

185,574 (score 12)

92,764 - 185,574 (score 10)

69,584 - 92,764 (score 6)

46,405 - 69,584 (score 4)

27,815 - 46,405 (score 3)

9276 - 27,815 (score 2)

<9276 (score 1)

Socio-economic status as per the Modified Kuppuswamy 2024 scale

26-29 Upper (I)

16-25 Upper middle (II)

11-15 Lower middle (III)

5-10 Upper lower (IV)

<5 Lower (V)

Education of the study participant

Professional degree/graduate/intermediate/diploma

High school

Middle school

Primary school

Illiterate

Occupation of the study participant

Working: Public sector or private sector

Not working: Retirement

Mention occupation:

Current source of income

Pensions

Job/profession

Interests/rent

Relatives

Friends

NGOs

None

Any other (specify)

Financial dependence - Independent/dependent

Section B: Morbidity Pattern

1.     Specify disease suffering from - …………………

2.     For how long have you been suffering from this condition?

………….(year)……(month)   ………….(year)……(month)   ………….(year)……(month)

3.     Staying with family / alone

4.     Preferred place of health seeking- Government/Private/Both

5.     Are you currently on medication for the same illness? (mention medications with doses, frequency)

6.     Number of medications - monotherapy/ dual therapy / polytherapy

7.     Where you buy medication from - public / private/ both

8.     Do you take medicines regularly? Yes/No

If no, specify reason

Socio-economic status/availability /accessibility/health literacy / out-of-pocket expenses/ family and community support / multiple comorbidities / mental health /environmental factors

Any side effects from medication/others (if others, then specify) ………………………..

9.     Who is the decision maker in the family for taking/giving treatment: Self /Son/Daughter/Wife/ Others (Specify)

10.  General Medication adherence scale - Always (1) / Mostly (2) / Sometimes (3) / Never (4) - {mark for each question}

             1.     Difficulty in remembering to take medications

             2.     Forgetting medications due to a busy schedule, travel and other events

             3.     Discontinuing medication when feeling well

             4.     Stop taking medication due to adverse effects

              5.     Stop taking medication without informing the doctor

              6.     Discontinuing medicines due to other medicines for an additional disease

              7.     Find it hassle to remember medications due to the medication regimen complexity

              8.     Missing medicine due to progression of disease and addition of new medicine in the last month

              9.     Altering medication regimen, dose, and frequency

             10.  Discontinuing medications because they are not worth the money

              11.  Finding it difficult to buy medicine because they are expensive

11.  How often are visits advised by your treating physician?

       How often do you go for your follow-up visits?

       If irregular, then mention the reason for it.

12.  Any family history of: diabetes mellitus II/ hypertension/stroke/ cancer/ chronic lung disease/ others?

13.  Do you consume alcohol?

·       Yes / no / Past consumer

·       If yes, then mention duration …………..

·       In which form …………….

·       Quantity ………………

·       Frequency ………………..

14.  Do you consume tobacco? Yes/no / Past consumer           

·       Yes / no / Past consumer

·       If yes, then mention duration …………….

·       In which form: Form - Non-smoked - (Mishri/chewable tobacco) / smoked tobacco)

·       Quantity ……………..

·       Frequency …………….

15.  How often do you exercise?

·       Yes/no

·       Type of exercise - cycling, walking, running, others (………….)

·       How many days in a week ………….

·       Duration of exercise hours……. Minutes……

·       Are you following advice given by the doctor - Always / sometimes/ never

16.  Dietary habits

·       Type of diet - Veg / Non-veg / Mixed

·       Are you following the dietary advice given by the doctor?

Always / sometimes / never

·       Reason for not following advice ? ……………….

General examination:

Height in cm-

Weight in kg-

BMI- ……….. (Kg/m^2^)

Hip circumference -

Waist circumference -

Waist to hip ratio -……

BP: …………..(mmHg)

RBS - …………. (mg/dl)
